# Manual reduction and splint fixation for distal radius fracture with dislocation: a case report

**DOI:** 10.3389/fsurg.2025.1555268

**Published:** 2025-03-11

**Authors:** Wensheng Zhu, Shuangqiang Tu, Hairui Zhu, Feng Shan

**Affiliations:** ^1^ Department of Orthopaedics, Children’s Hospital of Soochow University, Suzhou, China; ^2^ Department of Orthopaedics, Huanggang Hospital of Traditional Chinese Medicine Affiliated to Hubei University of Chinese Medicine, Huanggang, China

**Keywords:** radius, reduction, distal radioulnar joint, dislocation, fracture management

## Abstract

**Introduction:**

Distal radius fractures (DRF) are one of the most common fractures, accounting for approximately 20% of all fractures. DRF is frequently associated with distal radioulnar joint (DRUJ) dislocation, which may be initially overlooked due to subtle symptoms and imaging findings. This can lead to misdiagnosis and suboptimal treatment.

**Patient concerns:**

An elderly female patient presented with a distal radius fracture. Early clinical and imaging evaluations failed to identify a co-existing DRUJ dislocation.

**Diagnosis:**

The DRUJ dislocation was subsequently diagnosed after careful assessment, highlighting the need for a thorough examination in cases of DRF.

**Interventions:**

The DRUJ dislocation was managed with manual reduction followed by splint fixation. The patient was closely monitored throughout the treatment process.

**Outcomes:**

Following the intervention, the patient demonstrated significant functional recovery, with improvement in wrist mobility and reduction in pain.

**Conclusion:**

This case underscores the importance of early detection of DRUJ dislocation in patients with DRF to avoid misdiagnosis and prevent long-term wrist dysfunction. Timely and appropriate intervention can lead to substantial recovery.

## 1 Introduction

Distal radius fractures (DRF) are a relatively common type of fracture in clinical practice (1), accounting for approximately 20% of all fractures (2). DRF is frequently accompanied by distal radioulnar joint (DRUJ) dislocation, which is often masked by symptoms and imaging findings, complicating early diagnosis. Improper treatment may lead to severe wrist joint dysfunction (3). Currently, there is no consensus on the clinical treatment of DRF (4, 5). Some researchers suggest that satisfactory outcomes can be achieved with manual reduction and external fixation for distal radius fractures. For DRF accompanied by concurrent DRUJ dislocation, the majority of scholars advocate for surgical treatment (6, 7). This case report presents a remedial treatment involving manual reduction and small splint fixation for a missed diagnosis of DRUJ dislocation. To our knowledge, this method has not been previously reported for the treatment of chronic DRUJ dislocation. The presented approach effectively avoids iatrogenic trauma associated with surgical intervention. The small splint fixation, which does not cross the joint, allows for early active wrist exercises without restriction. This treatment offers a novel therapeutic option for patients with DRF complicated by chronic DRUJ dislocation. The aim is to offer evidence-based recommendations for treatment strategies in this unique clinical scenario.

## 2 Case presentation

A 59-year-old female patient was admitted to the hospital on July 20, 2024, one hour after falling while being pursued by a dog, resulting in pain, deformity, and limited mobility in her left wrist. Upon admission, a specialized examination revealed a “dinner fork” deformity in the left wrist joint, significant swelling, local tenderness, palpable bone crepitus, and limited wrist joint mobility. The interphalangeal joint mobility was preserved, and blood circulation and sensation in the fingertips were normal. The patient had a history of good health. Emergency digital radiographs (DR) showed fractures of the distal radius and the ulnar styloid process in the left wrist. The initial diagnosis was a Colles fracture of the left radius and a fracture of the left ulnar styloid process. After discussion with the medical team, the patient opted for conservative treatment, which involved manual reduction and splint fixation.

**Figure 1:**
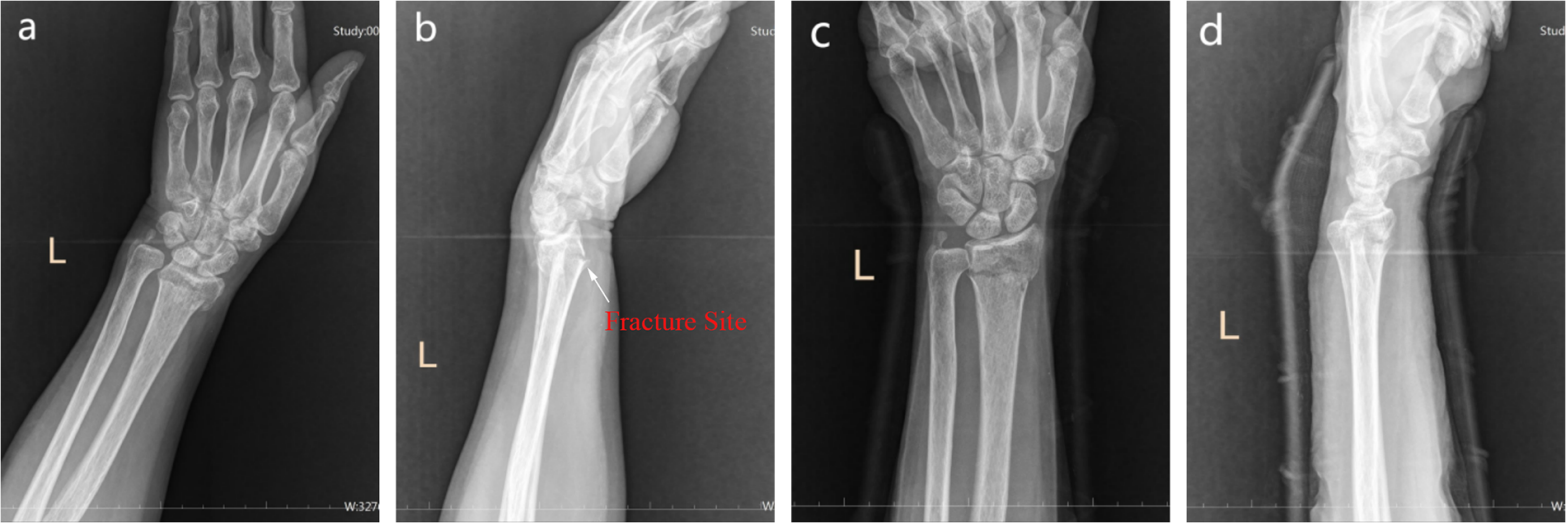
Anteroposterior and lateral wrist DRs from the emergency department and after reduction. **(a,b)** emergency DRs showing distal radius and ulna fractures, with radial dorsal displacement of the radius and a distal ulnar styloid fracture. **(c,d)** post-reduction DRs demonstrating satisfactory reduction of the radius, with evidence of DRUJ separation. DR, digital radiograph; DRUJ, distal radioulnar joint.

Upon admission, emergency manual reduction was performed using the following method: the patient's forearm was placed in a natural pronated position with the elbow flexed. An assistant held the forearm of the affected limb, while the operator's left hand held the patient's wrist, and the right hand grasped the base of the thumb on the affected limb, applying opposing traction. The operator then performed palmar flexion and ulnar deviation for reduction. A Colles splint was applied to the forearm and wrist joint, covering the palmar, dorsal, radial, and ulnar sides, and bandages were used for fixation. Immediate re-examination on DR imaging showed satisfactory restoration of the radial height, palmar tilt, and ulnar deviation angles (Figures 1a–d). The patient was given symptomatic treatment for swelling and pain relief and was instructed to perform active flexion and extension exercises of the finger joints.

**Figure 2:**
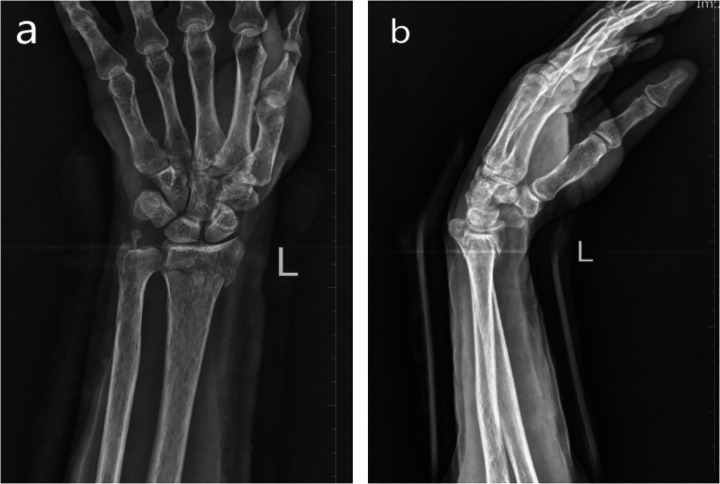
Anteroposterior and lateral wrist DRs at the two-week follow-up. **(a,b)** mild radial displacement of the distal radius with no abnormalities observed in the DRUJ. DR, digital radiograph; DRUJ, distal radioulnar joint.

Two weeks after reduction, after the swelling subsided, the splint became slightly loose. A follow-up DR showed good alignment and positioning of the distal radius, with slight radial displacement at the distal fracture site, and the splint was adjusted for tightness (Figure 2a,b).

**Figure 3:**
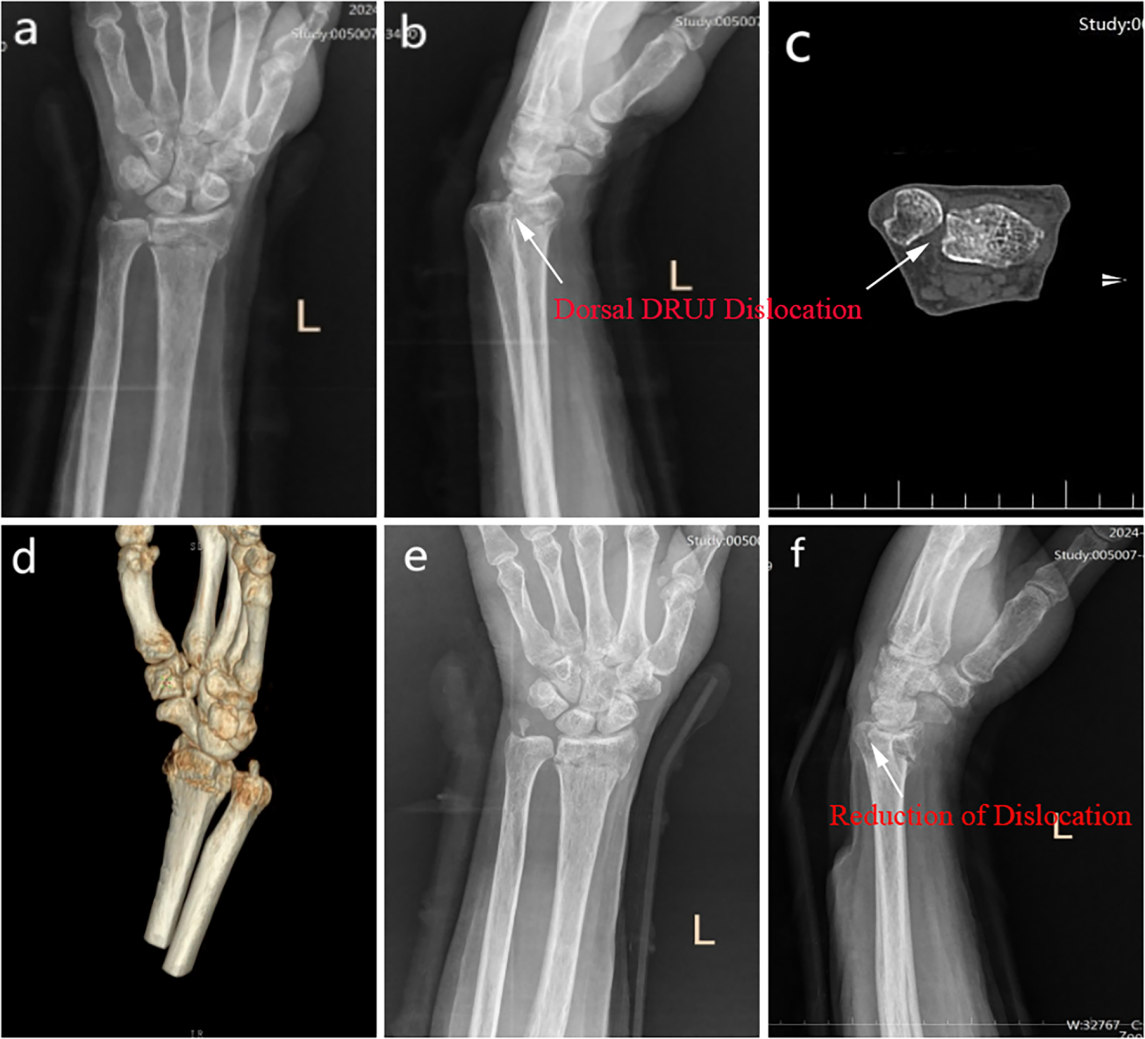
Drs and CT, and 3D reconstruction at the four-week follow-up. **(a–d)** no significant changes in the radius compared to previous imaging, with marked dorsal ulnar dislocation of the DRUJ. **(e–f)** the DRUJ is well-reduced, and the spacer is appropriately positioned. DR, digital radiograph; CT, Computed Tomography; DRUJ, distal radioulnar joint.

Four weeks after reduction, the patient reported ulnar-sided wrist pain, which subsided after performing flexion-extension exercises of the left hand. Follow-up DR and CT scans of the wrist showed no significant change at the radial fracture site, but there was dislocation of the DRUJ, with dorsal displacement of the ulnar head. The patient, fearing surgery, requested conservative treatment. Subsequently, a dorsal pad splint was applied with the forearm in a supinated position to stabilize the ulnar head. Immediate DR re-examination confirmed correction of the DRUJ dislocation (Figure 3a–f).

**Figure 4:**
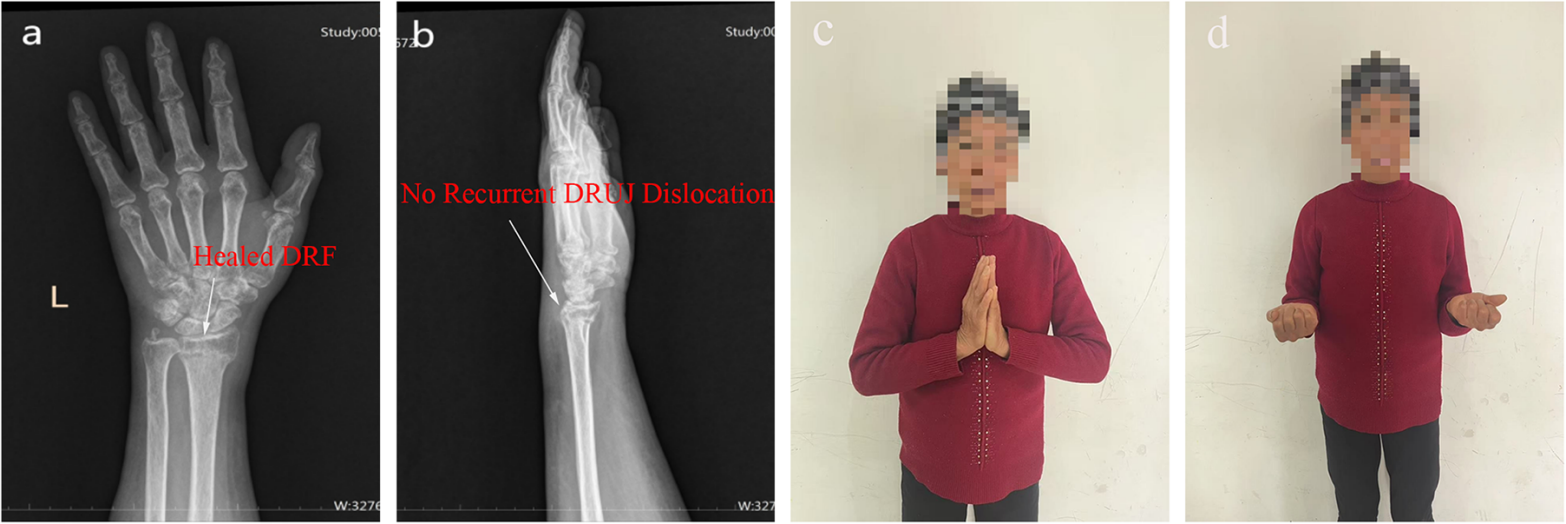
Two-month follow-up DRs after fixation. **(a,b)** satisfactory healing of the radius, with correction of the distal radioulnar joint dislocation. **(c,d)** the patient demonstrates good range of motion in wrist rotation and dorsiflexion. DR, digital radiograph.

Twenty-five days later, the splint was removed, and the patient was instructed to perform pronation and supination exercises of the forearm, as well as flexion and extension exercises of the wrist joint. A follow-up DR one week later showed satisfactory anatomical morphology of the distal radius, good fracture healing, restoration of the DRUJ anatomical relationship (Figure 4a–d), and good wrist joint function, with a Patient-Rated Wrist Evaluation score of 31 points.

## 3 Discussion

DRUJ dislocation is frequently overlooked in the treatment of DRF, with a missed diagnosis rate of up to 50% (8). Clinical manifestations include ulnar-sided wrist pain and restricted joint movement during forearm rotation (9). The present case presents a classic Colles fracture of the radius, with the ulnar fracture located at the tip of the styloid process, which did not require fixation (10). Initial emergency DR imaging showed signs of DRUJ dislocation, but due to concerns about positional effects during imaging, this was not initially emphasized. Fixation was performed with the forearm in a pronated position during the first reduction procedure, which may have contributed to the worsening separation of the DRUJ in the subsequent stages. Four weeks later, DRUJ dislocation was observed, at which point the dislocation had become chronic. Most scholars advocate for surgical repair in cases of chronic dislocation (6). In cases of dorsal DRUJ dislocation, the interosseous membrane is typically not injured. When the forearm is in a supinated position, the interosseous membrane and the pronator quadratus muscle become taut, preventing dorsal displacement of the ulnar head. The patient strongly preferred conservative treatment. Without surgery, a dorsal splint with the forearm in a supinated position was applied, with padding to stabilize the ulnar head. In the supinated position, tension in the palmar radioulnar ligament helped pull the dislocated ulnar head toward the palmar side, aiding realignment. With additional dorsal padding, a stabilizing force was applied to maintain DRUJ reduction, facilitating soft tissue repair around the DRUJ. For chronic DRUJ dislocation, surgical treatment typically involves repair or reconstruction of the TFCC complex, and in severe cases, even ulnar head replacement, which is associated with significant surgical trauma. In contrast, our approach using small splint fixation leverages the self-traction forces of the soft tissues around the wrist joint combined with the external fixation provided by the splint, maintaining the DRUJ in a dynamically stable state to achieve reduction. Although the patient in this case demonstrated good fracture healing, no recurrence of DRUJ dislocation, and satisfactory range of motion in forearm pronation, supination, wrist flexion, and extension at three months post-injury, the follow-up period was relatively short. It remains uncertain whether ulnar-sided wrist impingement and associated pain may develop in the long term. Further clinical observation over an extended period is necessary to evaluate these potential outcomes.

Based on this case study, the following key insights were summarized: (1) Clinicians must carefully examine imaging data, and if DRUJ dislocation is suspected in conjunction with distal radius fracture, wrist CT should be performed promptly to fully assess the injury (11); (2) When dorsal dislocation of the ulnar head is suspected, restoring the anatomical shape of the radius is essential for reduction, and fixation of the forearm in the supinated position after reduction is critical to maintaining the anatomical alignment of the DRUJ; (3) Early-stage fractures are at risk of re-displacement due to reduced bony support at the fracture site following bone resorption and weakened splint restraint as swelling subsides, necessitating timely adjustment of the splint tightness; (4) For confirmed fractures with DRUJ dislocation, surgical treatment is recommended.

## 4 Conclusion

This report presents a case of DRF complicated by DRUJ dislocation, which was successfully treated with manual reduction and splint fixation despite the missed initial diagnosis. Although this is merely a case study and not representative, it provides valuable insights into non-surgical treatment options for DRF complicated by DRUJ dislocation. This report aims to emphasize the importance of considering DRUJ injury when treating DRF.

## Data Availability

The original contributions presented in the study are included in the article/Supplementary Material, further inquiries can be directed to the corresponding author.
